# Unusual position of pilonidal sinus in children may explain its malformative etiology: Case report and review of the literature

**DOI:** 10.1016/j.ijscr.2024.109444

**Published:** 2024-02-24

**Authors:** Carmine Noviello, Mercedes Romano, Pierluigi Marzuillo, Ronchi Andrea, Alfonso Papparella

**Affiliations:** a Pediatric Surgery Unit, Department of Woman, Child, General and Specialized Surgery, University of Campania “Luigi Vanvitelli”, Naples, Italy; b Pediatrics Unit, Department of Woman, Child, General and Specialized Surgery, University of Campania “Luigi Vanvitelli”, Naples, Italy; c Pathology Unit, Department of Mental and Physical Health and Preventive Medicine, University of Campania “Luigi Vanvitelli”, Naples, Italy

**Keywords:** Pilonidal sinus, Face, Children

## Abstract

**Introduction:**

Pilonidal sinus is a condition that causes inflammation and abscesses in the sacral region and affects adolescents and young adults. The etiology of this condition remains controversial.

**Case presentation:**

A six year old boy was observed to have an orifice in the frontonasal region which contained hair. He had two previous infections which were treated with antibiotics. Magnetic Resonance Imaging showed no cranial malformations. Surgery was performed under general anesthesia and the pilonidal sinus was completely excised. At follow-up the child was in good health.

**Clinical discussion:**

This case in a child with a frontonasal skin anomaly highlights that skin anomalies may be a cause of pilonidal sinus.

**Conclusion:**

Skin malformations can be the underlying cause of pilonidal sinus in some cases.

## Highlights

• The Pilonidal Sinus is a disabling disease that mainly affects the skin region of the sacred in young adults. Its etiology is discussed. Many believe that the hair of the region associated with trauma and bad hygiene are the cause of infection.
• We report a case of pilonidal sinus in an atypical position that occurred with infection in the first year of life and that demonstrates the possibility of a skin malformation as a cause of the pilonidal sinus.
• This nasal malformations must be treated as soon as they present with a primary complete excision because of high risk of infection or intracranial complications.

## 1 Background

The term Pilonidal Sinus (PS) originates from the Latin words ‘pilus’ (hair) and ‘nidus’ (nest), and was first introduced by Hodges in 1880 to describe a skin condition in the region between the buttocks (1). Its main characteristic is the presence of hair inside, leading to recurrent inflammatory processes. This condition can be debilitating for young adults. While the pathology has been known for some time, its etiology remains unclear. Some consider it to be an acquired disease related to hair and inflammation in the sacrococcygeal area ([2], [3], [4]), while others believe that a malformation of the sacral region, resulting in the formation of a cyst or fistula, creates conditions for infection and inflammation (5,6). The pilonidal sinus is a common condition in the sacrococcygeal region, with a frequency of approximately 26 cases per 100,000 people (6). However, it is important to note that the characteristics of the disease, such as the presence of a fistula with hair inside, may also occur in atypical locations, including the face and midline (7).

The authors report a case of a child with a pilonidal sinus located in the frontonasal region. Surgery was required due to recurrent infections.

### 1.1 Methods

This is a case report from our pediatric surgical department, reported in accordance with the SCARE criteria (8).

### 1.2 Case report

**Fig. 1:**
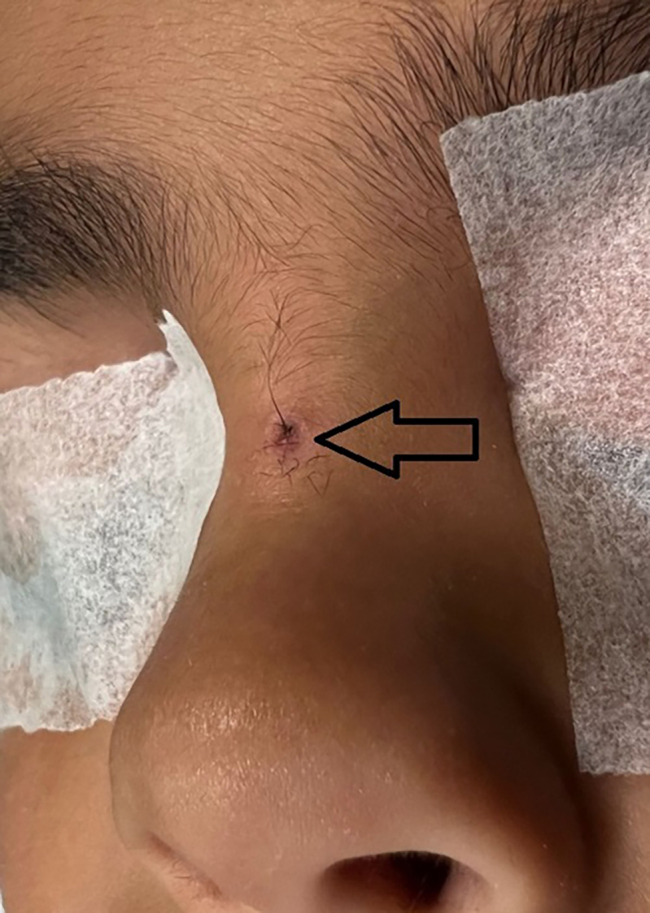
Preoperative image of the pilonidal sinus in front-nasal region: black arrow shows the pilus inside the fistula.

**Fig. 2:**
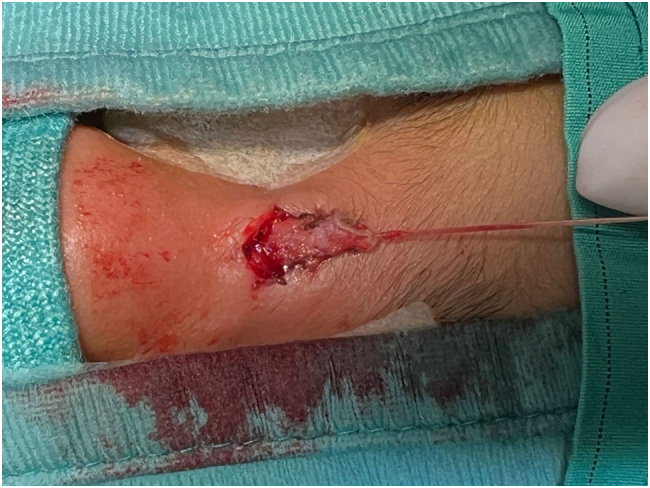
intraoperative image of the peripherical incision and removing of entire the fistula.

**Fig. 3:**
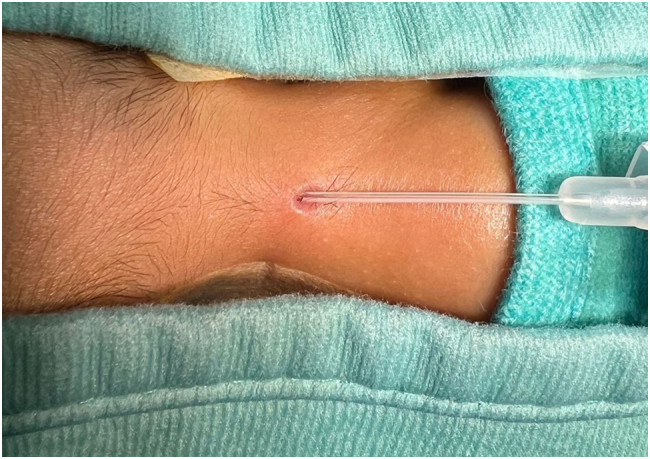
Image of probe inside the fistula for better removal.

**Fig. 4:**
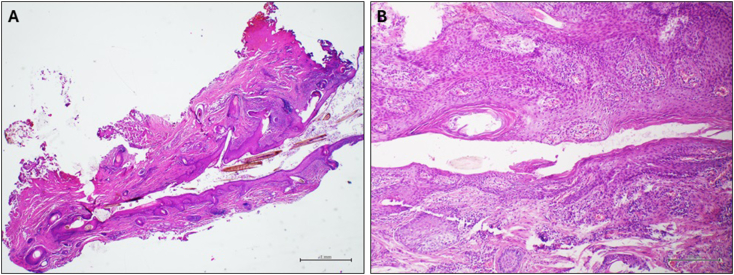
Histological examination showing a fistula that crosses the dermis containing hair shafts (A, haematoxylin and eosin stain, original magnification 20×). The fistula is lined by squamous epithelium with lumps of hair (B, haematoxylin and eosin stain, original magnification 100×).

**Fig. 5:**
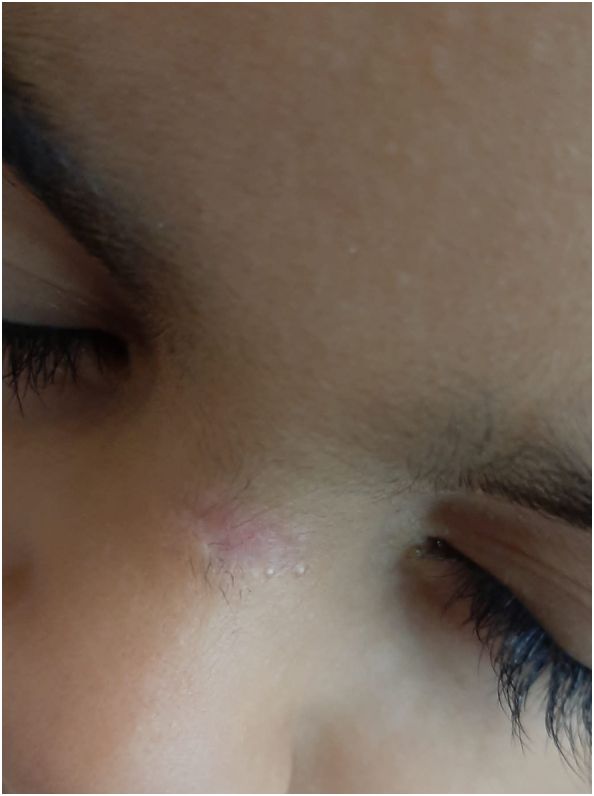
Image of the scar at 6-month follow up.

A six-year-old boy presented at our attention with an orifice in the frontonasal region containing hair (Fig. 1). The lesion had appeared at the age of six months, with hair protruding from the orifice and discharge with smelly fluid. The patient had twice infection with inflammation of the region and pus discharge, one when he was five-year-old and another one year later. In this case, the patient received antibiotic treatment for recurrent infections. Surgical excision of the PS was then indicated and the treatment performed when the infection was completely solved (three months after antibiotic treatment). Prior to surgery, a Magnetic Resonance Imaging (MRI) was performed on the child to verify the presence of a fistula with the skull. The MRI, which was performed with sedation, did not reveal any malformations. The surgery was conducted under general anesthesia with a peripheral incision at the fistula orifice (Fig. 2), which was completely removed with the help of a probe (Fig. 3). Aesthetic reconstruction of the wound was then performed. Histological examination allowed the diagnosis of dermoid cyst (Fig. 4). The child underwent clinical and ultrasound follow-up that showed no recurrence (Fig. 5).

## 2 Discussion

**Fig. 6:**
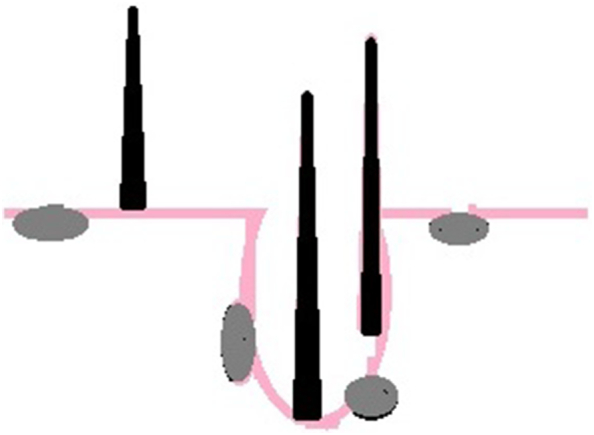
Schematic representation of pilonidal sinus: in black the hairs, in grey the glans.

The PS is a malformation that contains hairs and can cause infections and abscesses in the sacrum-coccygeal region. It mainly affects young adults with excessive hair growth and prolonged sitting, causing compressive trauma to the sacrum. According to some authors, its incidence is 26 per 100,000 people (6). It is generally believed that the pathology is acquired when hairs in the sacral fissure penetrate the skin, causing an inflammatory and foreign body reaction in the subcutaneous tissues, which may lead to abscess formation or chronic sinus formation. However, pediatric surgeons have observed cases of cutaneous fistula in the sacral region without signs of infection. In some cases the origin can be a skin malformation, in which a fistula is present from birth and then becomes deeper and tighter. The affected area contains glands and hair roots (Fig. 6). In cases of sacral localization, these may begin to grow during adolescence or adulthood. In cases of localization in the nasal region where eyebrows are present, hair may be visible in children, as happened in present case.

PS can occur in locations other than the sacral region, as has been known for some time (7). However, this is quite rare, occurring in approximately 2.2 % of cases (9). The affected sites include the axilla (10), the suprapubic and umbilical areas (11,12), the face (5), and other areas such as the clitoris, penis, neck, finger, scalp, scrotum, anus, and intermammary region. Other authors have reported cases of pilonidal sinus in the facial area of adults and support a theory of trauma and hair to explain the infectious process (13). Facial localization appears to be more frequent in males who have a beard and are subject to shaving, supporting the ingrown hair theory as the cause of abscess and pilonidal sinus. According to some Authors this nasal malformations (dermoid cysts or sinuses) must be treated as soon as they present with a primary complete excision because of high risk of infection or intracranial complications (14). The present case and others in the Literature (14) demonstrate that the pilonidal sinus and resulting infections were caused by a skin malformation in the eyebrow region that grew around and inside the fistula. The secretion of sweat glands also contributed to the smelly fluid. It is important to note that when a skin communication deepens, it can result in a frontonasal fistula with cerebrospinal fluid leakage (15). Diagnosis of a PS is quite easy and rarely needs imaging, however in case of nasal localization, an MRI should be performed to exclude any intracranial communication. The treatment of PS can be different and it depends of site, age and experience of the center. Non-surgical option is possible: some Authors used a crystallized phenol application with good results (16), some other used a cauterization, alcohol injection or a phenol solution. The surgical option is various: the complete excision with primary closure is reported from long time and indicate for smaller PS (17), but in other cases (primary closure impossible or with tension) various kinds of flaps are reported (V—Y flaps and rhomboid flaps) (18). Recently, many reports had good aesthetic and functional results with minimally invasive techniques (endoscopic or video-assisted ablation) ([19], [20], [21]). Although our center has significant experience in minimally invasive treatments for this pathology, especially in the sacral site, for this case we opted for a complete excision with primary closure due to the patient's age, the size of the PS (small) and the very delicate area in which it was present.

## 3 Conclusion

The etiology of PS is still debated. The hypothesis that hair presence is associated with a traumatic and hygienic mechanism for infection remains valid. However, it is also important to consider the possibility of a malformation with fistula and the presence of hair roots and glands inside. This last mechanism was the basis of the infection in our patient, who required complete removal of the fistula.

## Informed consent

Written informed consent was obtained from the patient's parents/legal guardian for publication and any accompanying images. A copy of the written consent is available for review by the Editor-in-Chief of this journal on request.

## Ethical approval

The institution exempts the case report from ethical approval.

## Funding

No funding was received.

## Author contribution

Noviello Carmine: Study conception and design, Drafting of the manuscript

Pierluigi Marzuillo: Data acquisition

Romano Mercedes: Analysis and data interpretation

Ronchi Andrea: data (histology) acquisition

Papparella Alfonso: Critical revision.

## Guarantor

The guarantor of the study is Prof Carmine Noviello.

## Declaration of competing interest

The authors declare that there is no conflict of interests.
